# The association between the health-related physical fitness and inhibitory control in preschool children

**DOI:** 10.1186/s12887-022-03163-y

**Published:** 2022-02-24

**Authors:** Yiyan Li, Tang Zhou, Yanhua Lu, Menghao Sang, Jiajia Liu, Xiaolong He, Minghui Quan

**Affiliations:** 1 School of Kinesiology, Shanghai University of Sport, Shanghai, China; 2 School of Physical Education and Health Science, Zhejiang Normal University, 321004 Jinhua, China; 3 Shanghai Frontiers Science Rearch Base of Exercise and Metabolic Health, Shanghai University of Sport, Shanghai, China

**Keywords:** Preschool children, Health-related physical fitness, Flanker task, Inhibitory control, Reaction time

## Abstract

**Background:**

Inhibitory control develops rapidly during the preschool stage, and development of inhibitory control in this period is significant for the healthy growth of the future. However, most studies paid more attention to children and adolescents in recent years, but less focus on preschool children. Therefore, the purpose of this study was to explore the association between the health-related physical fitness and inhibitory control in preschool children.

**Methods:**

This cross-sectional study was based on a baseline data from randomized controlled trial by cluster sampling(including 128 preschoolers, 70 boys, 58girls).The health-related physical fitness T-score (HPFT) was obtained by adding standard scores of six indicators: body mass index, handgrip strength, standing long jump, one-leg balance, 2 × 10 m shuttle run test, and 20 m shuttle run test. Inhibitory control was assessed using the flanker task and reflected by reaction time and accuracy.

**Results:**

A total of 120 preschoolers were included in the final statistical analysis. After adjusting the confounders, HPFT was associated with reaction time (β=-2.49 ms, 95%CI: -4.22, -0.75). Similarly, a negative association was observed between one-leg balance and reaction time (β=-12.04 ms, 95%CI: -18.94, -5.15), and between 20 m shuttle run test and reaction time (β=-23.28 ms, 95%CI: -37.00, -9.56). Compared with the participants in the lowest tertile, HPFT (β=-158.74, 95%CI: -309.63, -7.84), one-leg balance (β=-267.25 ms, 95%CI: -420.71, -113.79) and 20 m shuttle run test (β=-215.58 ms, 95%CI: -374.67, -56.49) were all negatively associated with reaction time of those in the highest tertile.

**Conclusions:**

Negative relationships between HPFT and RT of the inhibitory control were observed in preschoolers. To have better inhibitory control, it’s suggested that HPFT of preschoolers should be at least 249. These findings are of great significance for the early improvement of HPFT and the development of inhibitory control in preschool children.

**Supplementary Information:**

The online version contains supplementary material available at 10.1186/s12887-022-03163-y.

## Background

Executive functions (EFs), a subset of advanced cognitive functions that coordinate with each other to complete complex cognitive tasks, mainly include inhibitory control, working memory, and cognitive flexibility [1, 2]. Inhibitory control is the core of EFs, which refers to the ability of individuals to inhibit dominant behaviors unrelated to current tasks. People with good inhibitory control can overcome strong instinctive tendencies and resist external temptations by controlling attention, behaviors, thoughts, and/or emotions [3]. Generally, inhibitory control appears in the preschool stage, forming a key foundation for the development of advanced cognitive processes in adulthood [4]. Preschool stage is the golden period for the development of inhibitory control [5], the good development of inhibitory control is related to future intellectual development, academic performance and health status [6, 7]. Therefore, how to help the improvement of preschool children’s inhibitory control is significant for public health.

Health-related Physical Fitness (HPF) refers to the ability to engage in daily activities, which is an important indicator for evaluating physical and mental health [8]. HPF is composed of cardiorespiratory fitness, musculoskeletal fitness, motor fitness, and body composition [9]. Children with better HPF have higher attention system efficiency, prefrontal cortex activation and executive function[10, 11]. Previous studies have demonstrated that HPF is associated with inhibitory control, however, most studies paid more attention to children and adolescents [11, 12]. For preschool children with the rapid development of inhibitory control, the relationship between HPF and inhibitory control is rarely studied.

Therefore, this study explores the relationships between the components of HPF and inhibitory control in preschool children. The findings of this study may serve as a foundation for HPF to help the development of inhibitory control in the preschool stage. According to previous research [6], we hypothesized that HPF would be negatively associated with inhibitory control in preschool children.

## Methods

### Participants

This cross-sectional study was based on a baseline data from a randomized controlled trial in 2018, entitled ‘The effect and mechanism of aerobic exercise on EFs in preschool children: randomized controlled and imaging studies (ChiCTR1900021552)’. One hundred and twenty-eight preschool children (70 boys, 58 girls) from four kindergartens in Yangpu District, Shanghai, were recruited by cluster sampling. The inclusion criteria were as follows: (1) preschool children aged 3-6 years old; (2) physically healthy and no contraindications to exercise, such as cardiovascular diseases or neurological diseases; (3) voluntary participation (parents or legal guardian signed informed consent). This study was approved by the ethics committee of Shanghai University of Sport (ethics committee code: 2,017,023).

### Measurements

#### Basic information

The basic information of the participants was obtained through a questionnaire filled out by the participant's parents or legal guardians, including the participants’ age, sex, mother’s education (Below high school, Junior high school, Senior high school, College/Associate degree, Bachelor’s degree, and Master’s degree/Doctor’s degree), and household income (< 9,000RMB, 9,000-30,000RMB, 30,000-100,000RMB, > 100,000RMB), etc.

#### Measurement of HPF

The measurement of HPF was according to the Chinese National Physical Fitness Measurement Standards Manual-preschool children version (CPFS-preschool) [13], including cardiorespiratory fitness (20 m shuttle run test, 20mSRT), musculoskeletal fitness (handgrip strength and standing long jump), motor fitness (one-leg balance and 2 × 10 m SRT), and body composition (BMI, kg/m^2^). The specific test methods are as follows:

1. Cardiorespiratory fitness

Cardiorespiratory fitness reflects the integrated ability to transport oxygen to muscles for sustained physical activity(PA) in the cardiovascular and respiratory systems [14]. The prevailing test for cardiorespiratory fitness is 20 m SRT [6]. During the test, an adult tester led children to jog back and forth at an increasing speed (starting with a speed of 8.5 km/h and increasing 0.5 km/h every minute) with the music rhythm between the lines 20 m apart until they were too tired to reach the end line. The test was conducted once, and the total number of round trips was recorded.

(2) Musculoskeletal fitness

Musculoskeletal fitness is the ability to maintain movement through muscle contraction against resistance, including muscle strength, muscle endurance, explosive strength and muscle flexibility. [15]. It is commonly assessed by handgrip strength and standing long jump (upper and lower limbs, respectively) in preschoolers [16]. As for the handgrip strength test, children were naturally standing, and their arms on the test side were stretched out straightly about 10° apart from the body. T.K.K.5401 (Takei, Niigata, Japan) was used for the test, and the handgrip length was adjusted to the best position according to children’s hand length [17]. As for the Standing long jump, children’s feet open to the same width as shoulders, standing behind the marking line, bending their knees and swinging their arms to jump forward. The two tests were measured twice, and the maximum values were recorded in kilograms (precision of 0.1 kg) and centimeters (precision of 0.1 cm), respectively.

(3) Motor fitness

Motor fitness refers to physical fitness related to sports performance and motor skills, which is composed of speed/agility and balance [9]. One-leg balance is a reliable indicator of balance for preschool children [9]. The tester held the stopwatch, gave the starting order, and timed from the time the tested child lifted one foot from the ground till the lifted foot touched the ground. The speed/agile quality of preschool children was evaluated by 2 × 10 m SRT, which has been adopted in China since 2003[13]. Children were led by an adult tester, who ran as fast as possible from the starting point to the end point (10 m apart) back and forth. Testers used a stopwatch to time. The test was measured twice, the shortest time was recorded as a final result (precision of 0.1 s).

(4) Body composition

Firstly, the height and weight of the participants were measured by the CPFS-preschool, and BMI (BMI= kg/m^2^) and BMI scores (see Additional file 1) were calculated [13]. Second, BMI was graded according to the cut-off point established by the Chinese Working Group on Obesity for Children (CWGOC) [18]. The BMI cut-off points for overweight and obesity in Chinese adults recommended by CWGOC are 24 kg/m^2^ and 28 kg/m^2^ ( BMI_24_, BMI_28_ ). BMI cut-off points for preschoolers were inferred through the method of ‘cut-off point screening’ (see Additional file 2), and BMI were graded as normal, overweight and obesity.

#### Measurement of inhibitory control

Flanker Task can objectively evaluate inhibitory control in preschool children [19]. This study used the Fish flanker task (FFT)in E-Prime software to investigate the inhibitory control.

**Fig. 1:**
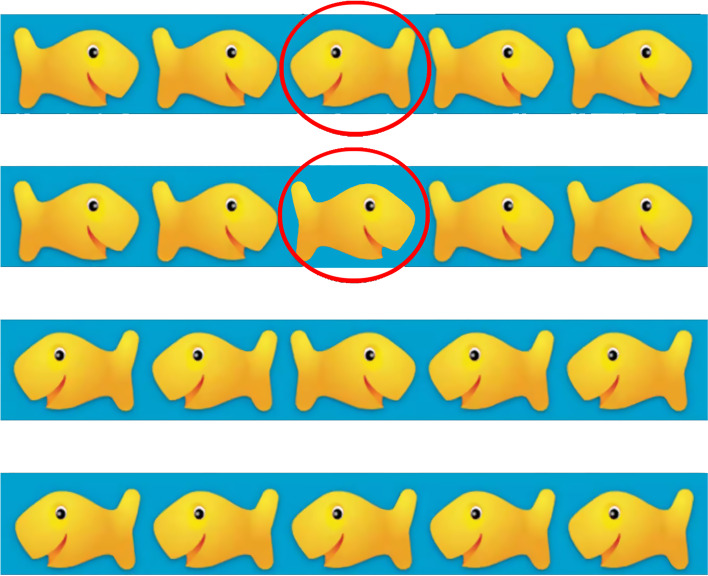
Fish Flanker Task

Before the formal test, there was a practice aimed to familiarize the participants with the test process, and require them to achieve more than 80% accuracy(ACC) before entering the formal test. The formal test was repeated 3 times, 40 judgments each time. Considering that the participants were younger (3–6 years old), they were guided by professionals during the test. The instructions were as follows: “There are five small fishes swimming in the water on the screen, but the middle fish is hungry. You can feed them with the button in your hand. When the middle fish swims to the left, you click the left button. When the middle fish swims to the right, you click the right button’’. At the beginning of the test, a set of stimulus pictures (Fig. 1) would appear randomly on the screen; pictures were divided into congruent conditions (the direction of all the fishes were the same) and incongruent conditions (the direction of the middle fish is different from the other fish). Participants immediately pressed the button after judging. If participants press the wrong button, or reaction time (RT) was less than 200 ms or more than 3 s, it would be judged as an invalid reaction, not included in statistical analysis. The formal test included 120 conditions (60 congruent conditions and 60 incongruent conditions). Two types of stimuli appeared at random with the same probability. The participants had a 10 s rest after every 40 judgments. The tests were completed one-on-one by an adult tester and participant in a quiet kindergarten classroom, and RT and ACC of each test were directly recorded on the software.

#### Statistical analysis

Continuous variables and categorical variables are presented as mean, standard deviation (SD), or percentages. The values beyond Mean ± 3SD are considered as outliers and removed from the final analysis. The scores of each index are converted into standardized scores with a mean of 50 and SD of 10. BMI was scored according to the CPFS-preschool. Because 2 × 10 m SRT is negatively associated with speed/agility, the score was multiplied by −1 and then standardized. The HPF standard score formula is T=[(X-M)/SD] × 10 + 50(X: personal performance, M: mean). The sum of each standard score was recorded as HPFT (T _standing long jump_ + T _handgrip strength_ + T _One−leg balance_ + T _2 × 10 m SRT_ + T _20 m SRT_ + T _BMI_).

Firstly, the relationship between HPFT and its four components with inhibitory control (ACC and RT) was tested by a multiple linear regression model. HPFT, T _standing long jump_, T _handgrip strength_, T _One−leg balance_, T _2 × 10 m SRT_, T _20 m SRT_ and T _BMI_ were categorized into tertiles ( T1 -T3, T1 defined as lowest tertiles ). With continuous variables and categorical variables as independent variables and inhibitory control as dependent variables, HPFT and its four components showed the relationship between ACC and RT after adjusting age, sex, mother’s education, and household income. Secondly, the piecewise linear regression was used to test whether there is a non-linear relationship and threshold effect between HPFT and inhibitory control. The R language-based Empower Stats software was used for statistical analysis *P *< 0.05 was considered statistically significant.

## Result

### Basic Information

**Fig. 2:**
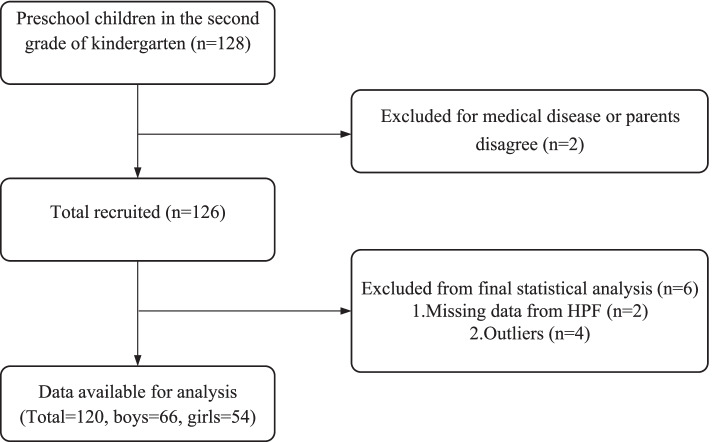
Flow of the participants screening in this study

A total of 128 preschool children were recruited from four kindergartens in Yangpu District. Two children were excluded because of inclusion criteria, and six children were excluded because of missing data or outliers. Finally, 120 children were included in the statistical analysis (66 boys and 54 girls, with an average age of 4.88 ± 0.38 years old) (Fig. 2).

The height, weight, and BMI of boys were significantly higher than girls, while there was no significant gender difference in demographic information such as mother’s education and household income. No significant gender difference was observed between HPFT and inhibitory control. Among the indicators of physical fitness, handgrip strength of boys was significantly higher than that of girls (*P* < 0.01), and one-leg balance of girls was significantly higher than that of boys (*P* < 0.01). In addition, other indicators had no significant difference between boys and girls (Table 1).

**Table 1:** Basic information for the participants

Characteristics	Boys(*n *= 66)	Girls(*n* = 54)	Total(*n *= 120)	*P* for sex
***Anthropometric characteristics***				
Age (years)	4.85 ± 0.36	4.92 ± 0.41	4.88 ± 0.38	0.324
Height (cm)	111.73 ± 4.95	109.57 ± 4.52	110.76 ± 4.86	**0.027**
Weight (kg)	20.40 ± 3.44	18.41 ± 2.40	19.50 ± 3.16	**0.002**
***Inhibitory control***				
ACC (%)	85.91 ± 13.68	85.32 ± 14.82	85.65 ± 14.15	0.752
RT (ms)	1236.14 ± 309.73	1249.35 ± 290.74	1242.09 ± 300.15	0.975
***Socioeconomic status***				
Mother’s education, n (%)				0.913
Below high school	2 (3.12%)	1 (2.08%)	3 (2.68%)	
Junior high school	6 (9.38%)	4 (8.33%)	10 (8.93%)	
Senior high school	12 (18.75%)	11 (22.92%)	23 (20.54%)	
College/associate degree	34 (53.12%)	27 (56.25%)	61 (54.46%)	
B.D.	10 (15.62%)	5 (10.42%)	15 (13.39%)	
M.D. / Ph.D.	2 (3.12%)	1 (2.08%)	3 (2.68%)	
Per capita household income (RMB/year)				0.731
< 9000	4 (6.25%)	4 (8.33%)	8 (7.14%)	
9000-30,000	13 (20.31%)	7 (14.58%)	20 (17.86%)	
30,000-100,000	19 (29.69%)	18 (37.50%)	37 (33.04%)	
> 100,000	28 (43.75%)	19 (39.58%)	47 (41.96%)	
***Body composition***				
BMI (kg/m^2^)	16.29 ± 2.14	15.29 ± 1.29	15.84 ± 1.87	**0.015**
Normal	47 (71.21%)	44 (84.62%)	91 (77.12%)	
Overweight	6 (9.09%)	8 (15.38%)	14 (11.86%)	
Obesity	13 (19.70%)	0 (0.00%)	13 (11.02%)	
***Musculoskeletal fitness***				
Handgrip strength(kg)	6.15 ± 2.18	4.88 ± 2.10	5.58 ± 2.23	**0.001**
Standing long jump (cm)	85.65 ± 15.68	85.34 ± 12.14	85.06 ± 14.15	0.555
***Motor fitness***				
One-leg balance(s)	10.25 ± 8.26	14.75 ± 9.79	12.27 ± 9.22	**0.003**
2 × 10 m SRT (s)	7.30 ± 0.80	7.35 ± 0.73	7.32 ± 0.77	0.642
***Cardiorespiratory fitness***				
20 m SRT (laps)	13.85 ± 4.67	13.19 ± 3.74	13.55 ± 4.27	0.430
*** HPFT***	299.62 ± 36.59	298.74 ± 32.86	299.22 ± 34.82	0.800

Continuous variables are resented as mean ± standard deviation, and classified variables are resented as percentage (%). Statically significant values are in bold

### Relationship between HPF and inhibitory control — results of multiple regression analysis

No significant interactions were found between HPFT and gender (P for interaction = 0.12), so boys and girls were combined for statistical analyses. There was a negative association between HPFT and RT in the multiple linear regression results (β=-2.49, 95%CI: -4.22, -0.75), after adjusting for age, sex, mother’s education, and household income. In addition, after adjusting confounding factors, for 1 s and 1 lap increased in one-leg balance and 20 m SRT, RT reduced by 12.04 ms (β = 12.04, 95%CI: -18.94, -5.15) and 23.28 ms (β = 23.28, 95%CI: -37.00, -9.56), respectively.

The results of categorized linear regression showed that when HPFT was divided into three groups (T1-T3, T1 was the lowest). Compared with the T1 group, RT of the T3 group reduced by 158.74 ms (β = -158.74, 95%CI: -420.71, -113.79). RT was negatively associated with one leg-balance (β = -267.25, 95%CI: -18.94, -5.15) and 20 m SRT (β = -215.58, 95%CI: -374.67, -56.49), after adjusted the confounding factors (Table 2).

**Table 2:** Associations of HPF and Inhibitory control in preschool children

HPF	Accuracy,*β* (95%CI)		Reaction time,*β* (95%CI)	
	**Model 1**	**Model 2**	**Model 1**	**Model 2**
**Musculoskeletal fitness**				
Handgrip strength (kg)	0.06 (-1.09, 1.21)	-0.80 (-2.01, 0.41)	-17.54 (-41.64, 6.56)	-17.76 (-46.27, 10.75)
Handgrip strength-tertile (kg)				
T1(1.00—4.50)	REF.	REF.	REF.	REF.
T2(4.60—5.75)	-0.96 (-7.64, 5.72)	-2.97 (-9.65, 3.70)	-65.54 (-206.98, 75.90)	-74.27 (-231.42, 82.87)
T3(6.00—12.00)	1.07 (-4.88, 7.02)	-3.34 (-9.72, 3.03)	-40.41 (-166.37, 85.55)	-39.76 (-189.78, 110.27)
*P for trend*	0.70	0.32	0.55	0.65
Standing long jump (cm)	0.11 (-0.07, 0.29)	0.03 (-0.15, 0.21)	-3.56 (-7.33, 0.21)	-3.51 (-7.60, 0.58)
Standing long jump-tertile (cm)				
T1(47.00—81.00)	REF.	REF.	REF.	REF.
T2(81.20—90.40)	5.93 (-0.23, 12.09)	2.59 (-3.81, 8.98)	-76.40 (-207.10, 54.30)	-62.52 (-211.50, 86.46)
T3(91.00—118.00)	3.03 (-3.13, 9.19)	-0.45 (-6.63, 5.72)	-124.56 (-255.26, 6.14)	-123.00 (-266.81, 20.81)
*P for trend*	0.34	0.86	0.06	0.10
**Motor fitness**				
One-leg balance (s)	0.14 (-0.13, 0.42)	0.09 (-0.22, 0.40)	**-8.62 (-14.28, -2.95)**	**-12.04 (-18.94, -5.15)**
One-leg balance-tertile (s)				
T1(1.80—7.60)	REF.	REF.	REF.	REF.
T2(7.70—13.00)	0.30 (-5.76, 6.37)	-0.37 (-6.41, 5.66)	-64.02 (-190.94, 62.90)	-78.86 (-215.49, 57.78)
T3(13.10—49.40)	7.34 (1.27, 13.41)	5.82 (-0.96, 12.60)	**-208.11 (-335.03, -81.19)**	**-267.25 (-420.71, -113.79)**
*P for trend*	0.02	0.11	**< 0.05**	**< 0.05**
2 × 10 m SRT (s−1)	2.93 (-0.34, 6.21)	0.66 (-2.71, 4.03)	-50.82 (-120.64, 19.00)	-53.42 (-132.07, 25.24)
2 × 10 m SRT-tertile (s^−1^)				
T1(-9.21—-7.58)	REF.	REF.	REF.	REF.
T2(-7.55—-6.90)	2.95 (-3.27, 9.17)	0.58 (-5.64, 6.80)	68.39 (-60.53, 197.31)	39.89 (-104.04, 183.83)
T3(-6.88—-5.80)	3.13 (-3.09, 9.35)	-0.52 (-7.12, 6.07)	-103.14 (-232.06, 25.78)	-95.65 (-248.24, 56.94)
*P for trend*	0.32	0.89	0.12	0.26
**Cardiorespiratory fitness**				
20 m SRT (laps)	0.58 (-0.01, 1.16)	0.18 (-0.44, 0.79)	**-21.90 (-33.96, -9.85)**	**-23.28 (-37.00, -9.56)**
20m SRT-tertile (laps)				
T1(5.00—10.00)	REF.	REF.	REF.	REF.
T2(11.00—13.00)	7.80 (1.13, 14.47)	4.39 (-2.91, 11.70)	-61.70 (-201.01, 77.62)	-56.11 (-221.69, 109.46)
T3(14.00—25.00)	6.07 (-0.20, 12.33)	1.46 (-5.56, 8.48)	**-193.71 (-324.49, -62.93)**	**-215.58 (-374.67, -56.49)**
*P for trend*	0.10	0.92	**< 0.05**	**< 0.05**
**Body Composition**				
BMI scores	-1.23 (-2.81, 0.36)	-2.04 (-3.58, -0.50)	4.11 (-29.79, 38.02)	6.25 (-31.07, 43.57)
**HPFT**	0.04 (-0.03, 0.12)	-0.02 (-0.10, 0.05)	**-2.25 (-3.75, -0.75)**	**-2.49 (-4.22, -0.75)**
**HPFT-tertile**				
T1 (218.45—280.85)	REF.	REF.	REF.	REF.
T2 (281.69—316.54)	4.49 (-1.70, 10.69)	2.10 (-4.30, 8.50)	23.90 (-103.98, 151.77)	-10.14 (-158.64, 138.37)
T3 (317.13—376.97)	2.25 (-3.95, 8.45)	-3.38 (-9.88, 3.12)	**-155.57 (-283.45, -27.70)**	**-158.74 (-309.63, -7.84)**
*P for trend*	0.48	0.30	**< 0.05**	**< 0.05**

Model 1: No Adjust. Model 2: Adjusting for age, sex, mother’s education, and household income. Statically significant values are in bold

### Relationship between HPF and Inhibitory control (RT)—piecewise linear regression

**Fig. 3:**
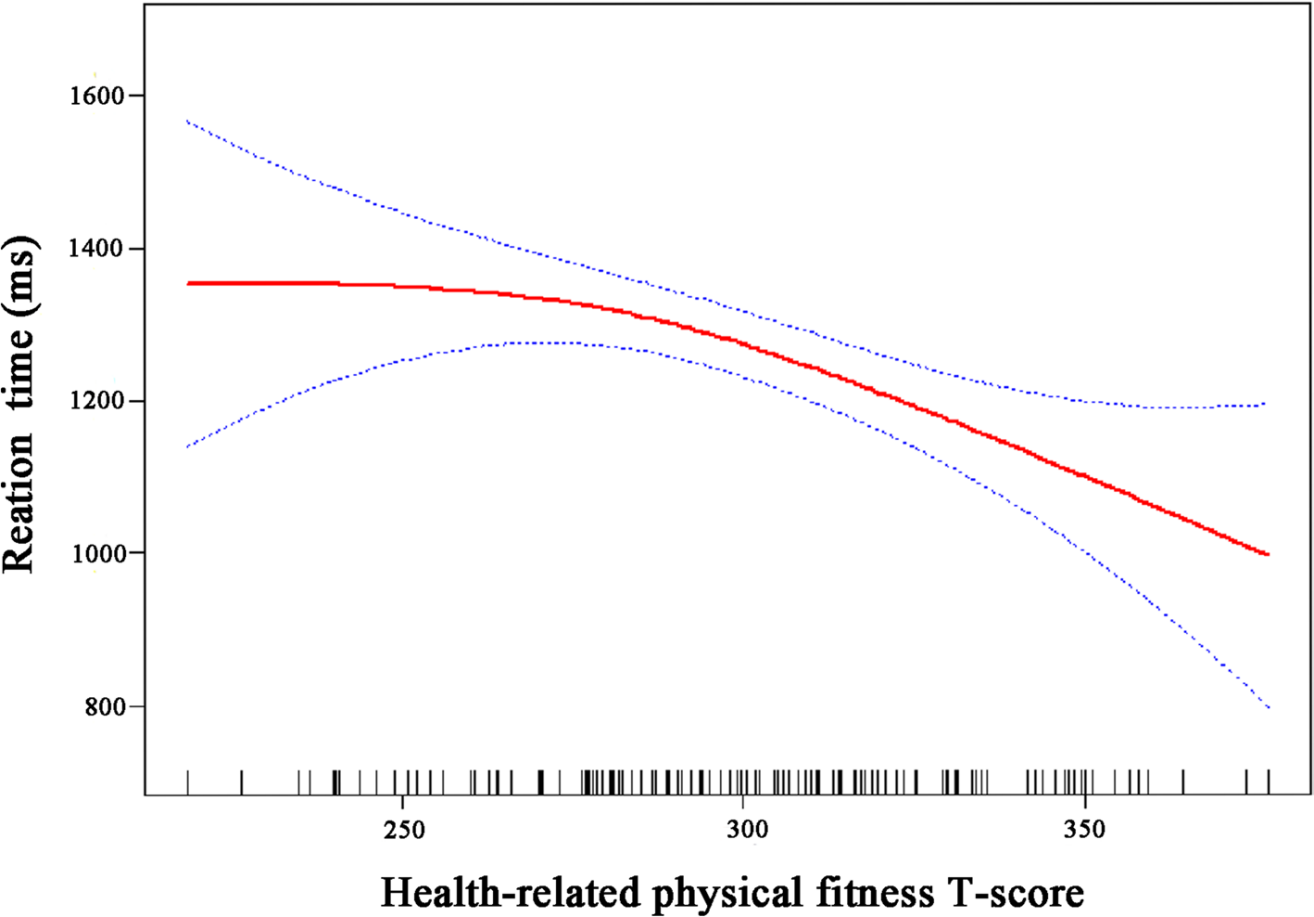
Association between HPFT and RT. The red solid line shows the fitted curves, and the blue dot lines show the 95% CI after adjusting for age, sex, mother’s education, and household income

Moreover, the Smooth curve fitting shows that there is a non-linear relationship between HPFT and RT in preschool children (Fig. 3). After adjusting age, sex, mother’s education, and household income, When HPFT is more than 249, for every 1 score of increase in HPFT, RT reduced by -3.46 ms (95%CI: -5.39, -1.53) (Table 3).

**Table 3:** Threshold effect analysis between HPFT and RT

HPFT	RT (β, 95%CI)	
	Model 1	Model 2
<249	1.92 (-1.93, 5.77)	13.36 (-1.36, 28.07)
>249	**-4.49 (-6.91, -2.08)**	**-3.46 (-5.39, -1.53)**
Likelihood Ratio	**0.021**	**0.026**

Model 1: No adjust. Model 2: Adjusting for age, sex, mother’s education and household income. Statically significant values are in bold

## Discussion

This study aimed to explore the association between HPFT and inhibitory control in preschool children. A negative relationship was observed between HPFT and RT in this study. Besides, one-leg balance and 20 m SRT were also negatively associated with RT. In addition, there was a nonlinear relationship between HPFT and RT. When HPFT was greater than 249, RT reduced significantly.

### Comparison of similar research results

The results of this study indicated that the higher HPF in the preschool stage was, the better RT was, consistent with the results of previous studies [20, 21]. Cardiorespiratory fitness was most concerned in HPF, which was considered as a significant predictor of inhibitory control [5]. High cardiorespiratory fitness in children is related to better EFs and differences in local brain structure and function [22]. Similar to other studies on children and adolescents, our study also found that preschool children’s cardiorespiratory fitness was significantly associated with RT [23]. However, most studies focus on children’s cardiorespiratory fitness and EFs, but few studies on preschool children. This study further enriches the evidence of such studies.

This study also found that a significant association between motor fitness and RT in preschool children, which was consistent with the results of Marion Stein. At the same time, Marion Stein believes that coordinated exercise can enhance inhibitory control [24]. Yu-Kai Chang’s research manifested that coordinated exercise intervention with different intensities has a positive effect on preschool children’s RT and ACC. In this study, the one-leg balance, an indicator for evaluating exercise adaptability, also appeared a negative association with RT. This may be because coordinated exercise changes the central cortex of the brain in the cortex and subcortex, and the central cortex involving the motor and sensory system matures earliest, thereby affecting cognition [25]. Long-term coordinated exercise may improve EFs by increasing the allocation of attention resources and accelerating the neural cognitive process [25]. In addition, similar results have been found in children and adolescents that motor fitness was associated with RT [26].

In addition, we observed no association between musculoskeletal fitness and inhibitory control, which is consistent with the results reported by Nieto-López’s study [5]. Although previous studies have proved that musculoskeletal fitness is beneficial to children’s health, including reducing obesity cardiovascular disease and metabolic risk factors [27], it possibily mainly related to strengthening cognitive inhibitory control and working memory [28]. At present, the existing evidence on musculoskeletal fitness and inhibitory control is insufficient, but some studies have covered that the relationships between musculoskeletal fitness and cognitive function. As proof, Reisberg’s cohort study found that preschoolers’ standing long jump to fat-free mass ratio was positively associated with perception (part of cognitive performance), but there was also no association between handgrip strength and cognitive performance[29]. Ruiz-Hermosa’s study also pointed out that children with better musculoskeletal fitness had better scores in verbal factors [30]. The inconsistency of these results may be affected by the differences in test methods and potential mixing factors [30, 31]. Therefore, it is necessary to continue to explore this aspect.

At the same time, no association was found in BMI scores and inhibitory control. Several studies agreed that obese children have significantly lower EFs than normal weight children [32]. However, in this study, the majority of participants with normal weight (77.12%) resulted in a smaller dispersion of BMI values, which may be the main reason for the lack of association between BMI and inhibitory control.

An interesting finding in this study was the non-linear relationship between HPFT and RT in preschool children. In other words, when HPFT was greater than 249, RT was significantly accelerated, and vice versa. It is indicated that the influence of HPF on inhibitory control may need to reach a certain threshold. Similarly, the association between HPF and academic performance had the same findings in the study of children and adolescents. For instance, the cardiorespiratory fitness of children in grade 2 and grade 3(aged 7.8 ± 0.6 years old) of primary school had a non-linear relationship with spelling and mathematics scores. When the score of the 20 m SRT was 22–28 laps, the cardiorespiratory fitness had a positive association with spelling and mathematics scores, but there was no significant association between them above 28 laps [33]. There were few reports on the level of HPT in preschool children to achieve the ideal inhibitory control level. Our findings of this study were an important supplement to this research problem.

However, this study did not find the relationship of HPT and its fitness components with ACC. In Marion Stein’s study, the improvement of motor fitness, especially the strengthening of coordination ability, would increase ACC [24]. This may be because FFT in this study was relatively simple, the overall accuracy was higher (85.65 ± 14.15%), to achieve the ceiling effect, so there was no significant relationship between the two. At present, few studies on HPT and ACC of preschool children, and the association between them needs to be further explored.

### Possible mechanism of the relationship between HPF and Inhibitory control

The reasons for the significant relationship between HPF and RT may be as follows. (1) Prior researches substantiated the belief that higher HPF level was possibly associated with regular moderate-to-vigorous intensity PA, and engaging in more PA has been confirmed beneficial to EFs by many studies [34]. Therefore, we speculate that HPF was related to inhibitory control because of students’ regular PA [6, 35, 36]. (2) Increased brain-derived neurotrophic factor (BDNF) and insulin-like growth factor 1 (IGF-1) in the prefrontal cortex triggered by PA, can regulate the network involved in executive function, thus affecting the development of inhibitory control [37]. (3) HPF is closely related to brain structure, and better HPF may promote the growth of brain structural cells, thereby assisting in the development of inhibitory control in the early life [38, 39].

### Strengths and limitations

This study has several strengths. Firstly, our research includes four main components of HPF, cardiorespiratory fitness, musculoskeletal fitness, motor fitness and body composition, and not only discusses the relationship between single component and inhibitory control, but also integrates each component of HPF to show its relationship more intuitively by using the form of standardized scores. Secondly, multiple linear regression and piecewise linear regression were used to reveal the non-linear relationship between HPF and inhibitory control.

Several limitations are also worth noting. First, this study used a cross-sectional study design, which cannot make the inference of causality. Second, a cluster sampling was recruited from Yangpu district, Shanghai, which may limit the popularization of our research results in the population. Third, the sample size of this study is relatively small, it is indispensable to increase the sample size and longitudinal studies to verify the relationship between HPF and inhibitory control in the future. Third, in addition to the known potential confounding factors that have been controlled, there might still have been other potential confounding factors that were not considered or measured.

### Significance for public health

The findings of this study may have the following implications:

1. Significant association were observed to HPF (including cardiorespiratory fitness and motor fitness) and inhibitory control in preschoolers. This finding provides a potential method for how to safely and effectively improve inhibitory control in the early stage of life.
2. In addition, we found a non-linear relationship in HPFT and inhibitory control when HPFT>249, which suggests that the HPFT may be used as one of the predictors to evaluate the development level of inhibitory control of preschoolers in the future. Since HPF is a safe, effective and low-cost test method, it is simple and feasible to implement in kindergartens. However, due to the inconsistency of the current research results, the establishment of this evaluation system needs to be further studied.

## Conclusions

In summary, we identified a significant association between health-related physical fitness and inhibitory control in preschool children, especially when health-related physical fitness T-score was greater than 249. At the same time, we observed that the better motor fitness and cardiorespiratory fitness, the better performance of inhibitory control. Therefore, improving health-related physical fitness of preschool children is more likely to benefit the development of inhibitory control. On this basis, health-related physical fitness T-score greater than 249 may be performed to a reference index to evaluate the development of inhibitory control, which is conducive to the future supervision of physical fitness and executive functions of preschool children. Subsequently, the relationships among health-related physical fitness, executive functions and other fitness components should be further verified through large-scale longitudinal studies and interventions.

## Supplementary Information

**Additional file 1. **Height standard weight of children aged 3-6 (boys). Height standard weight of children aged 3-6 (girls)

**Additional file 2. **BMI cut-off point of 3-6 years old preschool children in China

## Data Availability

The data and analysis during the current study are available from the corresponding author on reasonable request.

## Abbreviations

ACC: Accuracy
BMI: Body mass index
B.D.: Bachelor's degree
CPFS: preschool: Chinese National Physical Fitness Measurement Standards Manual-preschool children version
EFs: Executive functions
HPF: Health-related physical fitness
HPFT: Health-related physical fitness T-score
M.D: Master's degree
PA: Physical activity
Ph.D: Doctor's degree
RT: Reaction time
SRT: Shuttle run test
