# Supplementary material for: The association between the health-related physical fitness and inhibitory control in preschool children
Source: BMC Pediatr. 2022 Feb 24;22:106. doi: 10.1186/s12887-022-03163-y (PMC8867775; doi:10.1186/s12887-022-03163-y)
Supplement: Supplementary file 1 — Additional file 1. Height standard weight of children aged 3-6 (boys). Height standard weight of children aged 3-6 (girls) [file 12887_2022_3163_MOESM1_ESM.docx]

**Additional file 1.1** Height standard weight of children aged 3-6 (boys)

| Height segment(cm) | Weight (kg) | | | | |
| --- | --- | --- | --- | --- | --- |
|  | 1score | 3 score | 5score | 3 score | 1score |
| 76.0-76.9 | <8.6 | 8.6-9.3 | 9.4-11.7 | 11.8-12.4 | >12.4 |
| 77.0-77.9 | <8.7 | 8.7-9.5 | 9.6-11.8 | 11.9-12.5 | >12.5 |
| 78.0-78.9 | <8.9 | 8.9-9.7 | 9.8-11.9 | 12.0-12.6 | >12.6 |
| 79.0-79.9 | <9.1 | 9.1-9.8 | 9.9-12.1 | 12.2-12.8 | >12.8 |
| 80.0-80.9 | <9.2 | 9.2-10.0 | 10.1-12.3 | 12.4-12.9 | >12.9 |
| 81.0-81.9 | <9.4 | 9.4-10.1 | 10.2-12.5 | 12.6-13.1 | >13.1 |
| 82.0-82.9 | <9.6 | 9.6-10.2 | 10.3-12.7 | 12.8-13.3 | >13.3 |
| 83.0-83.9 | <9.8 | 9.8-10.4 | 10.5-12.9 | 13.0-13.5 | >13.5 |
| 84.0-84.9 | <10.0 | 10.0-10.5 | 10.6-13.1 | 13.2-13.8 | >13.8 |
| 85.0-85.9 | <10.1 | 10.1-10.7 | 10.8-13.3 | 13.4-14.0 | >14.0 |
| 86.0-86.9 | <10.3 | 10.3-10.9 | 11.0-13.6 | 13.7-14.2 | >14.2 |
| 87.0-87.9 | <10.5 | 10.5-11.1 | 11.2-13.8 | 13.9-14.5 | >14.5 |
| 88.0-88.9 | <10.7 | 10.7-11.3 | 11.4-14.0 | 14.1-14.7 | >14.7 |
| 89.0-89.9 | <10.9 | 10.9-115 | 11.6-14.3 | 14.4-14.9 | >14.9 |
| 90.0-90.9 | <11.1 | 11.1-11.7 | 11.8-14.5 | 14.6-15.2 | >15.2 |
| 91.0-91.9 | <11.3 | 11.3-11.9 | 12.0-14.7 | 148-15.4 | >154 |
| 92.0-92.9 | <11.3 | 11.3-12.1 | 12.2-15.0 | l5.1-15.6 | >15.6 |
| 93.0-93.9 | <11.7 | 11.7-12.3 | 12.4-15.2 | 15.3-15.9 | >15.9 |
| 94.0-94.9 | <Il1.9 | 1L.9-12.5 | 12.6-154 | 15.5-16.1 | >16.1 |
| 95.0-95.9 | <12.1 | 12.1-12.7 | 12.8-15.7 | 15.8-16.4 | >16.4 |
| 96.0-96.9 | <12.4 | 12.4-12.9 | 13.0-16.0 | 16.1-16.6 | >16.6 |
| 97.0-97.9 | <12.6 | 12.6-13.2 | 13.3-16.2 | 16.3-16.9 | >16.9 |
| 98.0-98.9 | <12.8 | 12.8-13.5 | 13.6-16.5 | 16.6-17.2 | >17.2 |
| 99.0-999 | <13.0 | 13.0-13.7 | 13.8-16.8 | 16.9-17.5 | >17.5 |
| 100.0-100.9 | <13.3 | 13.3-14.0 | 14.1-17.0 | 17.1-17.7 | >17.7 |
| 1010-101.9 | <13.5 | 13.5-14.3 | 14.4-17.3 | 17.4-18.0 | >18.0 |
| 102.0-102.9 | <l3.7 | 13.7-14.6 | 14.7-17.6 | 17.7-18.3 | >18.3 |
| 103.0-103.9 | <l3.9 | 13.9-14.9 | 15.0-17.9 | 18.0-18.6 | >18.6 |
| 104.0-104.9 | <14.1 | 14.1-15.2 | 15.3-18.2 | 18.3-18.9 | >18.9 |
| 105.0-105.9 | <14.4 | 14.4-15.6 | 15.7-18.5 | 18.6-19.3 | >19.3 |
| 106.0-1069 | <14.6 | 14.6-15.8 | 15.9-18.8 | 18.9-19.6 | >19.6 |
| 107.0-107.9 | <14.8 | 14.8-16.0 | 16.1-19.1 | 19.2-19.9 | >19.9 |
| 106.0-108.9 | <15.0 | 15.0-16.2 | 16.3-19.4 | 19.5-20.3 | >20.3 |
| 109.0-109.9 | <l5.3 | 15.3-16.5 | 16.6-19.9 | 20.0-20.7 | >20.7 |
| 110.0-110.9 | <15.6 | 15.6-16.8 | 16.9-20.2 | 20.2-21.0 | >21.0 |
| 111.0-111.9 | <15.9 | l5.9-17.1 | 17.2-20.5 | 20.6-21.4 | >21.4 |
| 112.0-112.9 | <16.2 | 16.2-17.4 | 17.5-20.9 | 21.0-21.9 | >21.9 |
| 113.0-113.9 | <16.5 | 16.5-17.7 | 17.8-21.3 | 21.4-22.2 | >22.2 |
| 114.0-1149 | <16.8 | 16.8-17.9 | 18.0-21.8 | 21.9-22.6 | >22.6 |
| 115.0-115.9 | <I7.1 | 17.1-18.1 | 18.2-22.1 | 22.2-23.1 | >23.1 |
| 116.0-116.9 | <I7.4 | 17.4-18.3 | 18.4-22.5 | 22.6-23.5 | >23.5 |
| 117.0-117.9 | <17.8 | 17.8-18.5 | 18.6-22.9 | 23.0-24.0 | >24.0 |
| 118.0-118.9 | <18.1 | 18.1-18.7 | 18.8-23.4 | 23.5-24.5 | >24.5 |
| 119.0-119.9 | <18.5 | 18.5-18.9 | 19.0-23.8 | 23.9-25.0 | >25.0 |
| 120.0-120.9 | <18.9 | 18.9-19.2 | 19.3-24.3 | 24.4-25.5 | >25.5 |
| 121.0-121.9 | <19.3 | 19.3-19.5 | 19.6-24.7 | 24.8-26.0 | >26.0 |
| 122.0-122.9 | <19.6 | 19.6-20.0 | 20.1-25.3 | 25.4-26.5 | >26.5 |
| 123.0-123.9 | <20.0 | 20.0-20.4 | 20.5-25.8 | 25.9-27.1 | >27.1 |
| 124.0-124.9 | <20.4 | 20.4-20.8 | 20.9-26.3 | 26.4-27.7 | >27.7 |
| 125.0-125.9 | <20.8 | 20.8-21.3 | 21.4-26.9 | 27.0-28.3 | >28.3 |
| 126.0-126.9 | <21.2 | 21.2-21.7 | 21.8-27.4 | 27.5-28.9 | >28.9 |
| 127.0-127..9 | <21.6 | 21.6-22.2 | 22.3-28.0 | 28.1-29.5 | >29.5 |

*Cited from The National Physical Fitness Measurement Standards Manual-Preschool Children Version in China.

**Additional file 1.2** Height standard weight of children aged 3-6 (girls)

| Height segment(cm) | Weight (kg) | | | | |
| --- | --- | --- | --- | --- | --- |
|  | 1score | 3score | 5 score | 3 score | 1 score |
| 76.0-76.9 | <8.9 | 8.9-9.0 | 9.1-11.6 | 11.7-12.9 | >12.9 |
| 77.0-77.9 | <9.0 | 9.0-9.1 | 9.2-11.8 | 11.9-13.1 | >13.1 |
| 78.0-78.9 | <9.1 | 9.1-9.3 | 9.4-12.0 | 12.1-13.2 | >13.2 |
| 79.0-79.9 | <9.3 | 9.3-9.5 | 9.6-12.2 | 12.3-13.3 | >13.3 |
| 80.0-80.9 | <9.5 | 9.5-9.7 | 9.8-12.4 | 12.5-13.5 | >13.5 |
| 81.0-81.9 | <9.7 | 9.7-10.0 | 10.1-12.6 | 12.7-13.7 | >13.7 |
| 82.0-82.9 | <9.9 | 9.9-10.2 | 10.3-12.8 | 12.9-13.9 | >13.9 |
| 83.0-83.9 | <10.1 | 10.1-10.4 | 10.5-13.1 | 13.2-14.1 | >14.l |
| 84.0-84.9 | <10.3 | 10.3-10.6 | 10.7-13.3 | 13.4-14.4 | >14.4 |
| 85.0-85.9 | <10.5 | 10.5-10.8 | 10.9-13.5 | 13.6-14.6 | >14.6 |
| 86.0-86.9 | <10.7 | 10.7-11.0 | 11.1-13.7 | 13.8-14.8 | >14.8 |
| 87.0-87.9 | <10.9 | 10.9-11.2 | 11.3-14.0 | 14.1-15.1 | >l5.1 |
| 88.0-88.9 | <11.1 | 11.1-11.4 | 11.5-14.2 | 14.3-15.3 | >15.3 |
| 89.0-89.9 | <11.3 | 11.3-11.6 | 11.7-14.4 | 14.5-15.6 | >15.6 |
| 90.0-90.9 | <115 | 11.5-11.8 | 11.9-14.7 | 14.8-15.8 | >15.8 |
| 91.0-91.9 | <11.7 | 11.7-12.1 | 12.2-14.9 | 15.0-16.1 | >16.1 |
| 92.0-92.9 | <11.9 | 11.9-12.3 | 12.4-15.2 | 15.3-16.3 | >163 |
| 93.0-93.9 | <12.1 | 12.1-12.5 | 12.6-15.4 | 15.5-16.6 | >16.6 |
| 94.0-94.9 | <12.3 | 12.3-12.7 | 12.8-15.7 | 15.8-16.8 | >16.8 |
| 95.0-95.9 | <12.5 | 12.5-13.0 | 13.1-15.9 | 16.0-17.1 | >17.1 |
| 96.0-96.9 | <12.7 | 12.7-13.2 | 13.3-16.2 | 16.3-17.4 | >174 |
| 97.0-979 | <13.0 | 13.0-13.4 | 13.5-16.5 | 16.6-17.7 | >17.7 |
| 98.0-98.9 | <13.2 | l3.2-13.7 | 138-16.7 | 16.8-18.0 | >18.0 |
| 99.0-99.9 | <13.4 | 13.4-13.9 | 14.0-17.0 | 17.1-18.2 | >18.2 |
| 100.0-100.9 | <13.6 | 13.6-14.2 | 14.3-17.3 | 17.4-18.5 | >18.5 |
| 101.0-101.9 | <13.9 | 13.9-14.4 | 14.5-17.6 | 17.7-18.8 | >18.8 |
| 102.0-102.9 | <14.1 | 14.1-14.7 | 14.8-17.9 | 18.0-19.1 | >19.1 |
| 103.0-103.9 | <14.3 | 14.3-14.9 | 15.0-18.2 | 18.3-19.5 | >19.5 |
| 104.0-104.9 | <14.6 | 14.6-15.2 | 15.3-18.5 | 18.6-19.8 | >19.8 |
| 105.0-105.9 | <14.8 | 14.8-15.5 | l5.6-18.8 | 18.9-20.1 | >20.1 |
| 106.0-106.9 | <15.1 | 15.1-15.7 | 15.8-19.1 | 19.2-20.4 | >20.4 |
| 107.0-107.9 | <15.4 | 15.4-16.0 | 16.1-19.4 | 19.5-20.8 | >20.8 |
| 108.0-108.9 | <15.6 | 15.6-16.3 | l6.4-19.8 | 19.9-21.1 | >21.1 |
| 109.0-109.9 | <15.9 | 15.9-16.6 | 16.7-20.1 | 20.2-21.5 | >21.5 |
| 110.0-110.9 | <16.2 | 16.2-16.9 | 17.0-20.5 | 20.6-21.8 | >21.8 |
| 111.0-111.9 | <l6.5 | 16.5-17.2 | 17.3-20.8 | 20.9-22.2 | >22.2 |
| 112.0-112.9 | <16.8 | 16.8-17.5 | 17.6-21.2 | 21.3-22.6 | >22.6 |
| 113.0-113.9 | <17.1 | 17.1-17.8 | 17.9-21.6 | 21.7-23.0 | >23.0 |
| 114.0-114.9 | <17.4 | 17.4-18.2 | 18.3-21.9 | 22.0-23.4 | >23.4 |
| 115.0-115.9 | <17.7 | 17.7-18.5 | 18.6-22.2 | 22.3-23.8 | >23.8 |
| 116.0-116.9 | <18.0 | 18.0-18.8 | 18.9-22.8 | 22.9-24.3 | >24.3 |
| 117.0-117.9 | <18.4 | 18.4-19.2 | 19.3-23.2 | 23.3-24.8 | >24.8 |
| 118.0-118.9 | <18.7 | 18.7-19.6 | 19.7-23.7 | 23.8-25.2 | >25.2 |
| 119.0-119.9 | <19.1 | 19.1-20.2 | 20.3-24.1 | 24.2-25.8 | >25.8 |
| 120.0-120.9 | <19.4 | 19.4-20.5 | 20.6-24.6 | 24.7-26.3 | >26.3 |
| 121.0-121.9 | <19.8 | 19.8-20.8 | 20.9-25.0 | 25.1-26.9 | >26.9 |
| 122.0-122.9 | <20.2 | 20.2-21.2 | 21.3-25.4 | 25.5-27.5 | >27.5 |
| 123.0-123.9 | <20.6 | 20.6-21.6 | 21.7-25.8 | 25.9-28.1 | >28.1 |
| 124.0-124.9 | <21.0 | 21.0-22.0 | 22.1-26.2 | 26.3-28.7 | >28.7 |
| 125.0-125.9 | <21.4 | 21.4-22.5 | 22.6-26.5 | 26.6-29.4 | >29.4 |
| 126.0-126.9 | <21.8 | 21.8-23.0 | 23.1-26.9 | 27.0-30.2 | >30.2 |
| 127.0-127..9 | <22.2 | 22.2-23.4 | 23.5-27.3 | 27.4-30.9 | >30.9 |

*Cited from The National Physical Fitness Measurement Standards Manual-Preschool Children Version in China.
