# Supplementary material for: The association between the health-related physical fitness and inhibitory control in preschool children
Source: BMC Pediatr. 2022 Feb 24;22:106. doi: 10.1186/s12887-022-03163-y (PMC8867775; doi:10.1186/s12887-022-03163-y)
Supplement: Supplementary file 2 — Additional file 2. BMI cut-off point of 3-6 years old preschool children in China [file 12887_2022_3163_MOESM2_ESM.docx]

**Additional file 2** BMI cut-off point of 3-6 years old preschool children in China

| Age(years) | boy | | girl | |
| --- | --- | --- | --- | --- |
|  | overweight | obesity | overweight | obesity |
|  | BMI_24_ | BMI_28_ | BMI_24_ | BMI_28_ |
| 3 | 16.8 | 18.1 | 16.9 | 18.3 |
| 3.5 | 16.6 | 17.9 | 16.8 | 18.2 |
| 4.0 | 16.5 | 17.8 | 16.7 | 18.1 |
| 4.5 | 16.4 | 17.8 | 16.6 | 18.1 |
| 5 | 16.5 | 17.9 | 16.6 | 18.2 |
| 5.5 | 16.6 | 18.1 | 16.7 | 18.3 |
| 6 | 16.8 | 18.4 | 16.7 | 18.4 |

*Cited from Li H et al.
